# Breathlessness dimensions association with physical and mental quality of life: the population based VASCOL study of elderly men

**DOI:** 10.1136/bmjresp-2021-000990

**Published:** 2021-11-05

**Authors:** Lucas Cristea, Max Olsson, David Currow, Miriam Johnson, Jacob Sandberg, Magnus Ekström

**Affiliations:** 1 Faculty of Medicine, Department of Clinical Sciences Lund, Respiratory Medicine and Allergology, Lund University, Lund, Sweden; 2 Faculty of Heath, University of Technology Sydney, Sydney, New South Wales, Australia; 3 Hull York Medical School, The University of Hull, Hull, UK; 4 Center for Primary Health Care Research, Department of Clinical Sciences in Malmö, Lunds Universitet, Lund, Sweden

**Keywords:** perception of asthma/breathlessness

## Abstract

**Background:**

Breathlessness is a multidimensional symptom prevalent in elderly affecting many aspects of life. We aimed to determine how different dimensions of breathlessness are associated with physical and mental quality of life (QoL) in elderly men.

**Methods:**

This was a cross-sectional, population-based analysis of 672 men aged 73 years in a Swedish county. Breathlessness was assessed using Dyspnoea-12 (D-12) and Multidimensional Dyspnoea Profile (MDP), and QoL using the Short Form 12 physical and mental scores. Scores were compared as z-scores across scales and analysed using multivariable linear regression, adjusted for smoking, body mass index and the presence of respiratory and cardiovascular disease.

**Results:**

Worse breathlessness was related to worse physical and mental QoL across all the D-12 and MDP dimension scores. Physical QoL was most strongly associated with perceptional breathlessness scores, D-12 total and physical scores (95% CI −0.45 to −0.30). Mental QoL was more strongly influenced by affective responses, MDP emotional response score (95% CI −0.61 to −0.48). Head-to-head comparison of the instruments confirmed that D-12 total and physical scores most influenced physical QoL, while mental QoL was mostly influenced by the emotional responses captured by the MDP.

**Conclusion:**

Breathlessness dimensions and QoL measures are associated differently. Physical QoL was most closely associated with sensory and perceptual breathlessness dimensions, while emotional responses were most strongly associated with mental QoL in elderly men. D-12 and MDP contribute complimentary information, where affective and emotional responses may be related to function, deconditioning and QoL.

**Key messages**

- Which breathlessness dimensions were associated with the physical and mental quality of life (QoL) in elderly men?
- Sensory and perceptual breathlessness dimensions were associated with physical QoL while emotional responses were associated with mental QoL.
- We show that different dimensions of breathlessness are associated with worse physical and mental QoL.

## Introduction

Breathlessness is a dominant symptom in cardiorespiratory disease and other severe illnesses^1^ and is prevalent in the population, affecting the everyday life of about 20% of elderly (aged >70) in the community.^2–5^ Chronic breathlessness^6^ often remains an invisible problem for healthcare professionals,^7^ even though it is an important cause of suffering, adverse events, health service utilisation and reduced function. It is strongly associated with poorer prognosis,^8^ anxiety, depression, fear^9^ and worse health-related quality of life (QoL).^2^

Breathlessness is a multidimensional symptom, influenced by different cognitive, physiological, psychological and environmental factors.^9 10^ To gain an understanding of the person’s experienced symptom, multiple dimensions of breathlessness need to be considered including its intensity, sensory quality (descriptors), unpleasantness/discomfort, emotional responses (such as anxiety, sense of depression or frustration) and its impact on QoL.^2^ Multidimensional measurement is therefore important to adequately capture changes in the person’s health status and treatment effects. The high prevalence of depression and anxiety—compounded by emotional responses to the symptom itself, may impair the person’s sense of self efficacy, function^11^ and further worsen QoL.^12 13^

Physical limitation from breathlessness, measured using the modified Medical Research Council (mMRC) score,^14^ has been associated with worse health-related QoL in adults, including both worse physical and mental QoL measured using the Short Form-12 (SF-12).^2 15^

Multiple dimensions of breathlessness can be assessed using the validated instruments Dyspnoea-12 (D-12)^16^ and the Multidimensional Dyspnoea Profile (MDP).^17^ Both instruments are widely used and MDP was recently validated in elderly people^18^ and used for patients with chronic obstructive pulmonary disease (COPD).^19^ QoL can be assessed using a number of validated tools including the generic SF-12.^20^ Neither the D-12 nor the MDP used the SF-12 (nor SF-36) in their validation studies.

Data are limited on breathlessness in the general population and in relation to QoL. Since this is a populational sample, we expected to find a lower prevalence of conditions and symptoms. In elderly patients’ symptoms are more common than at a younger age, and a study on a populational level can increase the knowledge of public health among elderly. Population studies have mainly used the mMRC scale, which relates to the activity or exertion required to induce breathlessness and not the severity of the breathlessness per se.^14^ No study has evaluated which dimensions of breathlessness that most strongly impact physical and mental QoL. This knowledge is important as different dimensions may have particularly strong effects on different outcomes including QoL, and thus be important treatment targets.^21^ We hypothesise that breathlessness dimensions associate differently with QoL, and that affective and emotional responses, such as anxiety and fear, most strongly influence mental QoL.

The primary aim of this study was to evaluate which dimensions of breathlessness, measured using D-12 and MDP, were most strongly associated with physical and mental QoL in elderly men in the general population. Second, we performed a head-to-head comparison between D-12 and MDP, to evaluate which of their corresponding dimension scores (D12 total/MDP A1; D12 physical/MDP perception; D-12 affective/MDP emotional) most independently and strongly captured physical and mental QoL.

## Methods

### Study design and population

This was a cross-sectional, population-based analysis of 73-year-old men in the VAScular disease and Chronic Obstructive Lung disease (VASCOL) study. The design and measurements of the VASCOL study has been detailed elsewhere.^22^ VASCOL included 1302 men aged 65 years in 2011–2012 who participated in screening for aortic aneurysm and who consented to participate in a longitudinal follow-up study. No patient-reported outcomes were assessed at baseline. In 2019, a postal survey including validated Swedish versions D-12,^16 23^ MDP^17 24^ and SF-12^20^ were sent to participants in the VASCOL study who were still alive and with a known address. The present analysis included participants who had available data on the evaluated D-12, MDP and SF-12 scores.

### Assessments

#### Descriptives

Self-reported variables included height (cm), weight (kg), smoking status (current, former or never-smoker), smoking exposure (years and mean number of cigarettes per day) and the presence of physician diagnosed conditions.^22^

#### Breathlessness

D-12 comprises 12 items (descriptors) each scored a 4-point scale of 0 (none), 1 (mild), 2 (moderate) or 3 (severe).^23^ The first seven items pertain to the physical domain (D-12 physical) of breathlessness, while the remaining five items pertain to the affective domain (D-12 affective). The range for D-12 total score is 0–36: 0–21 for the physical score; and 0–15 for the affective score. Higher D-12 scores indicate worse breathlessness.^23^ D-12 is validated to be completed as a postal questionnaire.^16^

The MDP comprises 11 items which are rated on 0–10 Numerical Rating Scales, divided into three domains that were evaluated: (1) the MDP A1 which is the total unpleasantness or discomfort of breathing; (2) the MDP perception score (range 0–60) which is the sum of A1 and the intensities of five sensory qualities (muscle work or effort; air hunger; chest tightness or constriction; mental effort or concentration and breathing a lot); and (3) MDP emotional response score (range 0–50) with is the sum of the intensities for each of five emotional responses (depression; anxiety; frustration; anger and fright). Higher MDP scores reflect worse breathlessness.^24^ MDP is also validated to be completed as a postal questionnaire.^17^

#### Quality of life

SF-12 comprises 12 items divided into a physical and a mental subscore with six questions and ranging 0–100 each. Higher scores indicate worse QoL.^20 22^ Questions regarding the physical QoL include limitations in activities, accomplishments and pain interference, while the mental score pertains to questions on accomplishments, energy, mood and social time.

The time period used in this study for all breathlessness and QoL instruments was ‘during the last 2 weeks’.^22^ The V.2 of SF-12 was used for the measurements.

### Statistical analyses

Characteristics of the participants at the time of the questionnaire (baseline) were tabulated. Representativeness of the participants compared with the underlying population sample was assessed by comparing characteristics at the initial assessment (2011–2012) between people who were included and not included in the present analysis. Sensitivity analysis for the included and excluded groups were analysed using χ^2^ test.

Associations for each breathlessness score for MDP (A1, perception and emotional response scores) and D-12 (total, physical and affective scores) with SF-12 physical and mental QoL scores were analysed using linear regression. MDP has a wider range (0–10) which makes it more suitable for linear regression compared with D-12 (0–3) in which each score corresponds to a category. MDP was designed to be used mainly as individual scales, to measure one or several dimensions depending on the research question while D-12 was constructed to yield a summary score to describe the overall level of breathlessness. Unadjusted and adjusted models were conducted for the potential confounders: body mass index (BMI), smoking status, pack-years of smoking, presence of cardiovascular disease (myocardial infarction, heart failure, valvopathies, atrial fibrillation or stroke) and respiratory disease (COPD, asthma or other lung disease). Confounders were selected based on previous literature^23 24^ and subject matter knowledge using a directed acyclic graph (DAG; www.dagitty.net). Multicollinearity was checked with a correlation matrix of the included variables. Adjustment for age and sex was not needed; all participants were men aged 73 years. Level of physical activity was not included in the main model, as it could mediate (through deconditioning) part of the effect of breathlessness on QoL. A sensitivity analysis also including physical activity was performed.

To compare the strength of associations between breathlessness dimensions and QoL, all breathlessness and QoL scores were log transformed to obtain a more normal distribution and converted into z-scores ([raw score − mean]/SD of the score). Z-scores are an established method to enable comparisons of scores across scales with different ranges. Normality of the breathlessness scores and outcomes were assessed using distribution plots. Estimates were expressed with 95% CIs. Estimates are interpreted as the mean change (in SDs) of the QoL score for each SD increase in the breathlessness score.

For head-to-head comparison of the associations with QoL between D-12 and MDP, regression models were conducted with the corresponding score from each instrument (D-12 total/MDP A1; D12 physical/MDP perception; and D-12 affective/MDP emotional, respectively), adjusting for the confounders. The estimates are interpreted as the strength of association independent of that of the corresponding score of the other instrument. Statistical analyses were performed using Stata V.16.0.

Written individual informed consent was obtained from all participants.

### Patient and public involvement

Patients or the public were not involved in the design, conduct, reporting or dissemination plans of our research.

## Results

### Participants

Out of the 1302 participants in the initial VASCOL population sample in 2011–2012, 1193 (92%) participants were still alive and had a known address in 2019. Out of these participants, 907 (76%) participated in the 2019 follow-up by returning the questionnaire. After exclusion of 235 participants due to missing data on D-12, MDP or SF-12, a total of 672 men aged 73 years were included in the analysis.

Participant characteristics are shown in table 1. Any breathlessness (score ≥1) was reported for mMRC by 97 (14%), for D-12 total by 196 (29%) and for MDP A1 by 189 (28%). Moderate to severe breathlessness, defined as MDP A1 ≥4, was reported by 29 (4%). Compared with people excluded due to missing data, included participants had fewer current smokers (11% vs 15%; p=0.015) and fewer morbidities such as diabetes mellitus (7% vs 13%; p<0.001), online supplemental table S1.

10.1136/bmjresp-2021-000990.supp1 Supplementary data

**Table 1:** Characteristics of 672 men aged 73 years from the general population

Factor	Value
Age, mean (SD)	73.2 (0.7)
BMI, mean (SD)	27.2 (3.9)
Ever smoked, n (%)	436 (65.7)
Missing	8 (1.2)
Pack-years of smoking, median (IQR)	5.4 (0–11.4)
Morbidities, n (%)	
Respiratory disease	58 (8.6)
Asthma	34 (5.1)
COPD	26 (3.9)
Other respiratory diseases	8 (1.2)
Cardiovascular disease	201 (29.9)
Angina pectoris	48 (7.1)
Atrial fibrillation	104 (15.5)
Heart failure	27 (4.0)
Myocardial infarction	66 (9.8)
Valvopathies	36 (5.4)
Diabetes mellitus	93 (13.8)
Rheumatological disease	31 (4.6)
Stroke	50 (7.4)
Breathlessness and QoL	
mMRC, n (%)	
0	447 (66.5)
1	97 (14.4)
2	54 (8.0)
3	26 (3.9)
4	32 (4.8)
Missing	16 (2.4)
D-12 total score, mean (SD)	1.66 (4.16)
D-12 physical score, mean (SD)	1.08 (2.52)
D-12 affective score, mean (SD)	0.57 (1.81)
MDP A1 unpleasantness score, mean (SD)	0.71 (1.37)
MDP perception score, mean (SD)	2.63 (6.48)
MDP emotional response score, mean (SD)	1.76 (5.09)
SF-12 physical QoL, mean (SD)	46.66 (9.03)
SF-12 mental QoL, mean (SD)	54.41 (8.93)

Data presented as mean (SD) or frequency (%). Percentages may not sum to 100 due to overlapping categories.

BMI, body mass index; COPD, chronic obstructive pulmonary disease; D-12, Dyspnoea-12; MDP, Multidimensional Dyspnoea Profile; mMRC, modified medical research council; QoL, quality of life; SF-12, Short Form-12.

### Breathlessness dimensions and QoL

Associations of different breathlessness dimensions with QoL are shown unadjusted (online supplemental table S2) and adjusted for confounders in table 2. Worse breathlessness was independently associated with worse physical and mental QoL, across all the D-12 and MDP scores. However, the strength of associations differed between the breathlessness dimensions. Physical QoL was most strongly associated with the D-12 scores and MDP A1. In contrast, mental QoL was most strongly associated with the MDP emotional response summary score (−0.50; 95% CI −0.57 to −0.43) and across the individual MDP emotional responses (table 2).

**Table 2:** Association for each breathlessness dimension with physical or mental quality of life assessed using the Short Form 12 (SF-12)

	Association with SF-12 physical score, adjusted* beta (95% CI)	Association with SF-12 mental score, adjusted* beta (95% CI)
D-12		
Total score	−0.38 (−0.46 to −0.31)	−0.37(−0.45 to −0.29)
Physical score	−0.38 (−0.45 to −0.30)	−0.36 (−0.44 to −0.28)
Affective score	−0.33 (−0.4 to −0.26)	−0.33 (−0.41 to −0.25)
MDP		
A1 unpleasantness score	−0.33 (−0.40 to −0.25)	−0.34 (−0.42 to −0.26)
Perception score	−0.30 (−0.37 to −0.22)	−0.32 (−0.40 to −0.24)
Emotional response score	−0.24 (−0.31 to −0.17)	−0.50 (−0.57 to −0.43)
Descriptors		
Muscle work or effort	−0.23 (−0.30 to −0.15)	−0.25 (−0.33 to −0.17)
Air hunger	−0.23 (−0.30 to −0.16)	−0.25 (−0.33 to −0.16)
Chest tightness or constriction	−0.21 (−0.28 to −0.14)	−0.27 (−0.35 to −0.19)
Mental effort or concentration	−0.21 (−0.28 to −0.14)	−0.27 (−0.35 to −0.19)
Breathing a lot	−0.17 (−0.24 to −0.10)	−0.21 (−0.29 to −0.13)
Emotional responses		
Depression	−0.17 (−0.24 to −0.09)	−0.55 (−0.61 to −0.48)
Anxiety	−0.21 (−0.28 to −0.14)	−0.51 (−0.59 to −0.44)
Frustration	−0.24 (−0.31 to −0.17)	−0.44(−0.51 to −0.36)
Anger	−0.18 (−0.25 to −0.10)	−0.41 (−0.49 to −0.34)
Fright	−0.21 (−0.28 to −0.14)	−0.46 (−0.54 to −0.39)

To be able to compare the strengths of the associations between the different scales, all scores were log transformed (to yield more normal distributions) and analysed as z-scores. Associations are by linear regression each breathlessness score separately, adjusted for confounders.

*Adjusted for the confounders smoking history, packet-years, BMI, respiratory diseases, and cardiovascular diseases.

BMI, body mass index; D-12, Dyspnoea-12; MDP, Multidimensional Dyspnoea Profile.

### Comparison of D-12 and MDP

To explore which of the instruments’ dimension scores that were most strongly and independently associated with the QoL outcomes, the corresponding scores from D-12 and MDP were evaluated concurrently in the same models ‘head-to-head’, as shown in table 3.

**Table 3:** Comparison between D-12 and MDP of associations with physical and mental quality of life, adjusted for confounders

Outcome	D-12 score	MDP score
SF-12 physical score	Total −0.32 (−0.42 to −0.21)	A1 −0.09 (−0.20 to 0.02)
	Physical −0.33 (−0.44 to −0.22)	Perception −0.06 (−0.16 to 0.04)
	Affective −0.30 (−0.39 to −0.21)	Emotional response −0.04 (−0.13 to 0.05)
SF-12 mental score	Total −0.27 (−0.39 to −0.14)	A1 −0.14 (−0.26 to −0.01)
	Physical −0.26 (−0.38 to −0.14)	Perception −0.13 (−0.25 to −0.01)
	Affective 0.01 (−0.08 to 0.11)	Emotional response −0.51 (−0.61 to −0.42)

Associations of corresponding scores of D-12 and the MDP, analysed concurrently in the same model, with physical and mental QoL. QoL was assessed using the SF-12 questionnaire. To be able to compare the strengths of the associations between the different scales, all scores were log transformed (to yield more normal distributions) and analysed as z-scores.

All models were adjusted for the confounders smoking history, packet-years, BMI, cardiovascular diseases, and respiratory diseases.

BMI, body mass index; D-12, Dyspnoea-12; MDP, Multidimensional Dyspnoea Profile; QoL, quality of life; SF-12, Short Form 12.

This analysis confirmed that physical QoL was most strongly associated with the D-12 scores. When adjusting for the corresponding D-12 score, the MDP A1 and subdomain scores no longer showed any independent association with physical QoL.

Mental QoL was most strongly associated with the MDP emotional response score, which was the strongest among all evaluated associations (−0.51; 95% CI, −0.61 to −0.42), table 3. Controlling for MDP emotional response, the D-12 affective score showed no independent association with mental QoL. The findings were similar in a sensitivity analysis also adjusting for the level of physical activity.

## Discussion

### Main findings

Worse breathlessness, across all dimensions measured using D-12 and MDP, was strongly associated with worse physical and mental QoL. These associations were independent of potential confounders including smoking, BMI and the presence of respiratory and cardiovascular disease. However, breathlessness dimensions influenced QoL differently. Physical QoL was most strongly related to sensory and perceptual dimension scores most strongly captured by D-12. In contrast, mental QoL associated most strongly with emotional responses to breathlessness captured by the MDP.

### What this study adds

This is the first study to incorporate D-12 and MDP into the health assessment of the participants in a general population sample. We expected to find a lower prevalence of breathlessness in our populational sample. The findings extend those of the cross-sectional study by Currow *et al* that worsening disability from chronic breathlessness (measured using mMRC) was associated with increasingly impaired physical and mental QoL in an adult population, majority of the adults were non-smokers aged between 15–44 years.^2^

Morélot-Panzini *et al* also showed high correlates of MDP-A2 with SF-12 mental component and HADS in patients with COPD.^25^ We now show that different dimensions of breathlessness are associated with worse physical and mental QoL, and that the strengths of the associations differ between breathlessness dimensions and by type of QoL. D-12 scores were more strongly associated with the physical QoL, while emotional responses to breathlessness measured using the MDP most strongly influenced mental QoL. This is the first head-to-head comparison of D-12 and MDP, showing that the instruments contribute complementary information. D-12 captured both physical and mental factors—in line with the aim, development and multidimensional nature of the instrument^26^—while MDP most strongly captured affective and emotional responses strongly linked to mental QoL. This analysis also showed that when analysing physical QoL with D-12 and mental QoL using MDP, respectively, measuring and adding the corresponding score from the other instrument added no further independent information in terms of their association with QoL.

The present findings are in line with report that breathlessness catastrophising (exaggerated negative cognitive-emotional orientation towards an actual or anticipated noxious stimuli) was related to poorer QoL in adults with cystic fibrosis,^27^ and that depression, pain and a less constructive emotional response to breathlessness are unique predictors of poorer health-related QoL. This might have therapeutic implications as, like in pain catastrophising, a more adverse affective and emotional response to breathlessness (such as in breathlessness catastrophising) may undermine the response to the interventions^28^ including to opioids.^29^ High scores of emotional responses might be related to less effective mental coping which could be measured with Coping Scale for Adults.

### Strengths and limitations

Strengths of this study include the use of validated and established multidimensional breathlessness instruments, the relatively large sample size and available data that allowed several potentially important confounders to be addressed in the analysis. Another strength is that SF-12 V.2 is a valid instrument with independent subscales, which are not correlated with one another as shown by Ware *et al*.^30^

A limitation of this study is the fact that only 73-year-old men were studied, they were only included because only men participated in the VASCOL study. While this effectively avoids any bias related to sex and age, generalisability for women and other age groups may be limited, and future follow-ups in the VASCOL study is planned to also include women and younger age groups.^22^ Also, most participants were relatively healthy and few reported severe breathlessness, which is to be expected in a population sample like this. The prevalence of COPD and asthma is slightly lower but like other studies of men and women in the same age, and as expected the prevalence of COPD is higher in our study than studies of younger age groups.^31 32^ Suggested next steps are to evaluate the impact of different breathlessness dimensions in people with more severe underlying disease and severe breathlessness.

### Implications

The present findings have several implications. First, the differing associations with QoL support the importance of measuring multiple dimensions of breathlessness and that D-12 and MDP contribute complementary information. Moreover, the instruments may be more useful for capturing certain aspects of breathlessness and QoL, as shown for D-12 in terms of physical QoL, and MDP emotional responses which most strongly captured influence on mental QoL. In relation to these associations with QoL, adding measuring with the other instrument (D-12 or MDP) did not contribute any independent information—but this is likely to vary depending on the aim of the measurement and the outcome.

The present findings can be interpreted to support the hypothesis that certain dimensions—especially stronger affective and emotional reactions, are more likely to induce behavioural adaptation, leading to the vicious circle of inactivity, deconditioning and worsening symptoms.^33 34^ For research, our findings can be relevant when selecting the appropriate instruments and scales in studies of breathlessness and QoL. The reliability and usefulness of the instruments for symptom monitoring in clinical practice is unknown. Further studies are also needed on the impact of different breathlessness dimensions on other outcomes, such as self-efficacy and mental coping with chronic breathlessness, function and on the effect of symptomatic treatments.

## Conclusion

Across all dimensions, worse breathlessness is associated with worse physical and mental QoL. Physical QoL was most strongly correlated with sensory and perceptual dimensions scores, most strongly captured by D-12, whereas MDP emotional responses of breathlessness most strongly influenced mental QoL in elderly men in the population.

## Data Availability

All data relevant to the study are included in the article or uploaded as online supplemental information.
