# Supplementary material for: Breathlessness dimensions association with physical and mental quality of life: the population based VASCOL study of elderly men
Source: BMJ Open Respir Res. 2021 Nov 5;8(1):e000990. doi: 10.1136/bmjresp-2021-000990 (PMC8573661; doi:10.1136/bmjresp-2021-000990)
Supplement: Supplementary data [file bmjresp-2021-000990supp001.pdf]

Table S1. Comparison between people included and excluded in the analysis.

**Baseline characteristic at age 65**	**Included in this analysis**	**Excluded due to missing data on variables in the returned survey**	**Excluded as did not participate in the survey**
N	672	235	395
Body mass index, mean (SD) (kg/m^2)	28.2 (4.1)	27.9 (3.8)	28.5 (4.4)
FEV_1, mean (SD) (%)	3.2 (0.6)	3.2 (0.5)	3.1 (0.6)
FVC, mean (SD) (l)	4.2 (0.7)	4.2 (0.7)	4.1 (0.8)
Smoking status			
Current smoker	72 (10.7%)	19 (8.1%)	77 (19.5%)
Former smoker	379 (56.4%)	135 (57.4%)	193 (48.9%)
Never smoked	221 (32.9%)	81 (34.5%)	125 (31.6%)
Packet years of smoking, mean (SD)	21.4 (18.2)	18.6 (16.1)	27.1 (22.8)
Angina pectoris			
Yes	41 (6.1%)	13 (5.5%)	31 (7.8%)
No	620 (92.3%)	218 (92.8%)	357 (90.4%)
Unknown	10 (1.5%)	4 (1.7%)	6 (1.5%)
Missing	1 (0.1%)	0 (0.0%)	1 (0.3%)
Stroke			
Yes	18 (2.7%)	5 (2.1%)	19 (4.8%)
No	648 (96.4%)	228 (97.0%)	375 (94.9%)
Unknown	5 (0.7%)	2 (0.9%)	0 (0.0%)
Missing	1 (0.1%)	0 (0.0%)	1 (0.3%)
Myocardial infarction			
Yes	34 (5.1%)	10 (4.3%)	30 (7.6%)
No	631 (93.9%)	221 (94.0%)	358 (90.6%)
Unknown	6 (0.9%)	3 (1.3%)	6 (1.5%)
Missing	1 (0.1%)	1 (0.4%)	1 (0.3%)
Diabetes mellitus			
Yes	48 (7.1%)	31 (13.2%)	53 (13.4%)
No	620 (92.3%)	203 (86.4%)	340 (86.1%)
Unknown	4 (0.6%)	0 (0.0%)	1 (0.3%)
Missing	0 (0.0%)	1 (0.4%)	1 (0.3%)
Asthma			
Yes	34 (5.1%)	13 (5.5%)	24 (6.1%)
No	634 (94.3%)	217 (92.3%)	363 (91.9%)
Unknown	3 (0.4%)	4 (1.7%)	7 (1.8%)
Missing	1 (0.1%)	1 (0.4%)	1 (0.3%)

Abbreviations: FEV_1, forced expiratory volume in 1 second; FVC, forced vital capacity.

Table S2. Crude association for each breathlessness dimension with physical or mental quality of life (QoL) assessed using the Short Form 12 (SF-12)

	**Association with physical QoL<br>Beta (95% CI)**	**Association with mental QoL<br>Beta (95% CI)**
**D-12**		
Total score	-0.44 (-0.51 to -0.38)	-0.32 (-0.39 to -0.25)
Physical score	-0.44 (-0.50 to -0.37)	-0.31 (-0.38 to -0.24)
Affective score	-0.38 (-0.44 to -0.31)	-0.30 (-0.37 to -0.23)
**MDP**		
A1 unpleasantness score	-0.40 (-0.46 to -0.33)	-0.31 (-0.38 to -0.23)
Perception score	-0.38 (-0.44 to -0.31)	-0.29 (-0.36 to -0.22)
Emotional response score	-0.29 (-0.36 to -0.22)	-0.47 (-0.54 to -0.41)
**Descriptors**		
Muscle work or effort	-0.28 (-0.35 to -0.21)	-0.26 (-0.33 to -0.18)
Air hunger	-0.29 (-0.37 to -0.22)	-0.24 (-0.31 to -0.17)
Chest tightness or constriction	-0.27 (-0.34 to -0.20)	-0.26 (-0.33 to -0.19)
Mental effort or concentration	-0.27 (-0.34 to -0.20)	-0.27 (-0.34 to -0.20)
Breathing a lot	-0.25 (-0.32 to -0.18)	-0.21 (-0.29 to -0.14)
**Emotional responses**		
Depression	-0.20 (-0.27 to -0.13)	-0.53 (-0.59 to -0.46)
Anxiety	-0.24 (-0.31 to -0.16)	-0.51 (-0.57 to -0.44)
Frustration	-0.29 (-0.36 to -0.22)	-0.40(-0.47 to -0.34)
Anger	-0.22(-0.29 to -0.15)	-0.37 (-0.44 to -0.30)
Fright	-0.24 (-0.31 to -0.16)	-0.46 (-0.53 to -0.39)

To be able to compare the strengths of the associations between the different scales, all scores were log transformed (to yield more normal distributions) and analyzed as z-scores. Associations are analyzed for each breathlessness score separately using uni-variable linear regression.
